# Musculoskeletal disorders among doctors and nursing officers : an occupational hazard of overstrained healthcare delivery system in western Rajasthan, India

**DOI:** 10.1186/s12891-023-06457-z

**Published:** 2023-05-04

**Authors:** Diksha Mahajan, Manoj Kumar Gupta, Neha Mantri, Nitin Kumar Joshi, Sridevi Gnanasekar, Akhil Dhanesh Goel, Srikanth Srinivasan, Nitesh Manohar Gonade, Suresh Kumar Sharma, Mahendra Kumar Garg, Pankaj Bhardwaj

**Affiliations:** 1 School of Public Health, All India Institute of Medical Sciences Jodhpur, Jodhpur, Rajasthan India; 2 Department of Community Medicine & Family Medicine, All India Institute of Medical Sciences Jodhpur, Jodhpur, Rajasthan India; 3 Department of Physical Medicine and Rehabilitation, All India Institute of Medical Sciences, Jodhpur, Rajasthan India; 4 Department of Medicine, All India Institute of Medical Sciences Jodhpur, Jodhpur, Rajasthan India

**Keywords:** Musculoskeletal Disorders, Doctors, Nursing officers, Ergonomic, Risk factors, Occupational Health, Physical activity

## Abstract

**Background:**

The present study was conducted to estimate the prevalence and distribution of MSDs in different anatomical regions among Doctors and NO and to determine their ergonomic risk factors and predictors.

**Methods:**

This cross-sectional study was conducted in an apex institution in Western India. The socio-demographic information, medical and occupational history, and other personal and work-related attributes were captured using a semi-structured questionnaire, which was developed and finalized by piloting on 32 participants (who were not part of the study). Nordic Musculoskeletal and International Physical Activity Questionnaires were used to assess MSDs and Physical activity. Data were analyzed using SPSS v.23. Prevalence of Musculoskeletal Symptoms (M.S.), Multisite Musculoskeletal Symptoms (MMS), and Widespread Musculoskeletal Symptoms (WMS) were calculated. A comparison was made to estimate the burden and distribution of MSD among Doctors and Nursing officers. Logistic regression was applied to identify the predictors of MSDs and pinpoint the risk factors associated with MSDs.

**Results:**

A total of 310 participants, of which 38.7% were doctors, and 61.3% were Nursing Officers (NOs) were included in the study. The mean age of the respondents was 31.63 ± 4.9 years. Almost 73% (95%CI: 67.9–78.1) of participants had MSD in the last 12 months, with approximately 41.6% (95%CI: 36.1–47.3) suffering from MSDs in the previous seven days of the survey. The lower back (49.7%) and the neck (36.5%) were the most affected sites. Working in the same position for a long time (43.5%) and not taking adequate breaks (31.3%) were the highest self-reported risk factors. Females had significantly higher odds of having pain in the upper back [aOR:2.49(1.27–4.85)], neck [aOR:2.15(1.22–3.77)], shoulder [aOR:2.8 (1.54–5.11)], hips [aOR:9.46 (3.95–22.68)] and knee [aOR:3.8(1.99–7.26)].

**Conclusions:**

Females, who are NOs, work for > 48 h per week, and fall in the obese category were significantly at more risk of developing MSDs. Working in an awkward position, treating an excessive number of patients in a day, working in the same position for a long period, performing repeated tasks, and not having enough rest breaks were significant risk factors for MSDs.

**Supplementary Information:**

The online version contains supplementary material available at 10.1186/s12891-023-06457-z.

## Background

Musculoskeletal Disorders (MSDs) are frequently characterized by pain, discomfort, and numbness that primarily affect the joints, bones, muscles, spine, and multiple body areas, leading to limitations in the affected area’s mobility, dexterity, and functioning [1, 2]. These ultimately cause decreased productivity, increased stress and poor sleep quality [3, 4]. This also leads to increased Disability-Adjusted Life Years (DALYs) and a high economic burden to the individual and society [5].

Musculoskeletal Disorders (MSDs) are known to be multifactorial. They can be attributed to personal characteristics (prior medical history, physical capacity, aging, smoking, obesity, etc.) or work-related attributes (high work demand, lack of control over work, low job satisfaction, repetitive work, the high pace of work, time pressure, lack of support and physical exposure like lifting, carrying, pulling, pushing, repetition of movements, awkward or cramped position and static posture, prolonged standing, and sitting, etc.) [5–7].

The World Health Organization (WHO) estimates that there are 1.71 billion MSD patients globally [1]. Data from the WHO’s Global Health Observatory (GHO) on the number of medical doctors per 10,000 populations, which showed a significant disparity, ranging from 7.35 in India to 26.01 in the UAE, 32.74 in France, and 7.35 in Sweden (70.92) [8]. Additionally, India’s nursing and midwifery professionals ratio is lower (1.7/1000 population) than the usual WHO guidelines of 3 nurses per 1000 people [9]. These figures represent the overburdened healthcare delivery system in India. In such overstrained working situations, Doctors and Nursing Officers are also prone to various occupational risk factors for developing MSDs. This is a new public health concern gradually shaping into an epidemic. Fortunately, it is preventable through good ergonomic practices. However, before designing intervention, a baseline estimate of the burden, risk factors, treatments, and existing preventative strategies is necessary.

Published literature has reported the wide variations in the prevalence of MSDs among healthcare workers s in different parts of India [10–14]. This may be attributed to variations in the risk factors and predictors of MSDs in different geographical locations in the country. Still, there is a need to explore the regional burden of MSDs among Doctors and Nursing officers involved in the direct care of the patients in our study region and their risk factors to adopt appropriate and timely interventions. The present study was conducted to estimate the prevalence and distribution of MSDs in various anatomical regions among doctors and nursing officers and determine the ergonomic risk factors and predictors of those MSDs. Besides that, the study also intended to compare the burden and distribution of MSD among Doctors and Nursing Officers in the Western Rajasthan.

## Methods

This cross-sectional study was conducted at an apex institution in Western India. The data collection was from June 2021 to August 2021. Study participants included the HCPs (Faculties, Senior Residents, and Nursing Officers). Those with autoimmune disorders and musculoskeletal trauma within one year due to any reason (e.g., accidents) were excluded from this study.

**Fig. 1:**
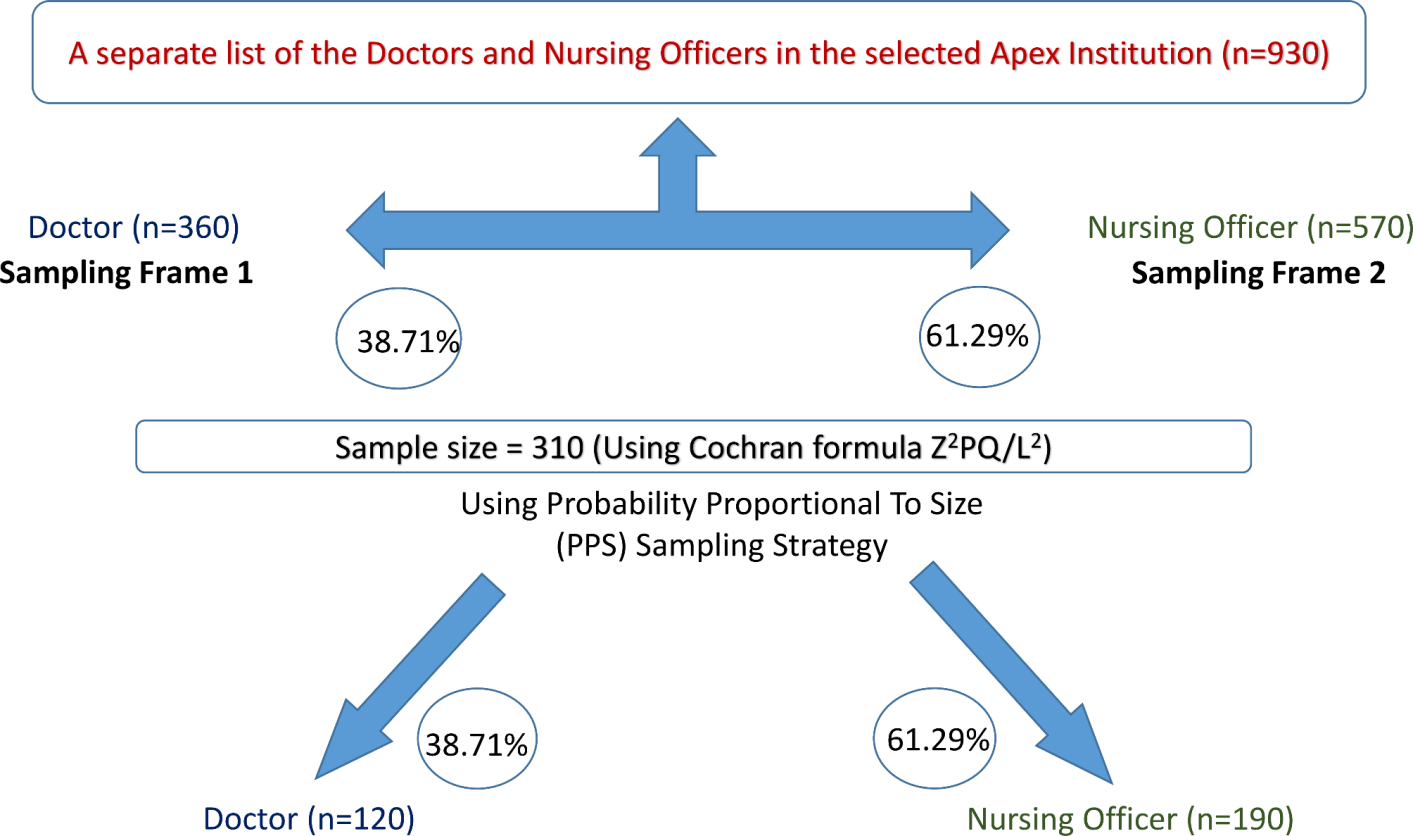
Sampling strategy

After assuming the midpoint (58%) from the range of prevalence of MSDs among healthcare workers (40–75%) quoted by previous studies [6, 10–14] in India, a permissible level of error at 10% and a non-response rate of 10%, the sample size was calculated using the formula z^2^pq/l^2^ (Cochran’s formula). The final sample size came out to be 310 participants. The lists of all the doctors and Nursing Officers (NOs) working in the Institution were prepared. The sample size for each group was fixed using Probability Proportion to Size (PPS) sampling. The participants were ultimately enrolled using simple random sampling. Face-to-face interviews were conducted with the participants for 25–30 min. The sampling strategy is shown in the flowchart (Fig. 1).

A semi-structured interview schedule was developed and piloted on 32 participants who were not part of the study. The information about the socio-demography, medical and occupational history, personal and work-related attributes and risk factors for MSD were captured using this questionnaire.

*Nordic Musculoskeletal Questionnaire (NMQ)*, a standardized instrument used to analyze musculoskeletal symptoms in an ergonomic or occupational health context, was used to assess MSDs [15]. This is already validated in the Indian population to determine which body regions are affected by musculoskeletal problems [16]. This questionnaire evaluates general health problems related to the musculoskeletal system at nine different positions on the body (neck, shoulder, upper back, elbow, wrist/hands, lower back, hip/thigh, knees, and ankle/foot) during the last 12 months and within the last seven days (point prevalence).

*International Physical Activity Questionnaire (IPAQ*) was used to gather information about physical activity. A short form of output was used to score the physical activity in categories (low activity levels, moderate activity levels, or high activity levels) [17].

### Definition of evaluation criteria for MSDs

- *Musculoskeletal symptoms (MS)* have been defined by dividing them into two categories; during the past 12 months and in the last seven days.
- *Multisite Musculoskeletal symptoms (MMS) were* defined by the presence of MS declared by the participants on two or more anatomical sites among the nine anatomical sites.
- *Widespread Musculoskeletal symptoms (WMS)* were described as musculoskeletal symptoms of the upper limb (shoulder/upper arm, elbow/forearm, wrist/hand, and neck), lower limb (hip/thigh, knee/lower leg, and ankle/foot), and axial (upper back and lower back) by American College of Rheumatology (ARC) [18].

The study was approved by Institutional Ethics Committee (Ref. AIIMS/JDH/IEC/2021/3498). Written consent was obtained from the participant after explaining the purpose of the study before administering the questionnaire. Data were analyzed using SPSS v.23. Prevalence of MS, MMS, and WMS was calculated using descriptive statistics at each site. The Chi-square was used for univariate analysis. Binomial logistic regression was applied to identify the predictors of MSDs and pinpoint the risk factors associated with MSDs. P value < 0.05 was considered statistically significant.

## Results

A total of 310 participants, of which 38.7% were doctors and 61.3% were Nursing Officers (NOs), were included in the study. The mean age of the respondents was 31.63 ± 4.9 years. There was a male predominance (71.0%) among the study participants. Most of them (76.1%) were married. Thirty-one (10.0%) participants were addicted to smoking or alcohol. As much as 70.3% of the HCPs were either overweight or obese. Almost half of the doctors and three fourth (76.9%) of the NOs had at least five years of work experience. The mean working hours per week for doctors and NOs were 53.7 ± 16.8 and 44.0 ± 8.5, respectively (Table 1).

**Table 1:** Socio-demographic characteristics and work-related attributes of the HCPs

Variables	Doctor (n = 120)	NOs (n = 190)	Total (n = 310)
	No. (%)	No. (%)	No. (%)
**Age group (years)**			
< 30	22 (18.3)	92 (48.4)	114(36.7)
30–39	80 (66.7)	96 (50.5)	176(56.7)
≥ 40–49	18 (15.0)	2 (1.1)	20(6.4)
Mean ± SD	34.35 ± 5.7	29.9 ± 3.3	31.63 ± 4.9
**Sex**			
Male	78 (65.0)	142 (74.7)	220(71.0)
Female	42 (35.0)	48 (25.3)	90(29.0)
**Marital status**			
Unmarried	27 (22.5)	47 (24.7)	74(23.8)
Ever Married	93 (77.5)	143 (75.3)	236(76.1)
**Addictions**			
Smoking	9 (7.5)	4 (2.1)	13(4.1)
Alcohol	11 (9.2)	7 (3.7)	18(5.8)
**Body mass index**			
< 18.5	3(2.5)	4 (2.1)	7(2.2)
18.5–22.9	31 (25.8)	54 (28.4)	85(27.4)
23-24.9	26 (21.6)	59 (31.0)	85(27.4)
> 25	60 (50.0)	73 (38.4)	133(43.0)
Mean ± SD	25.3 ± 4.2	24.3 ± 3.1	24.7 ± 3.6
**Years of experience**			
< 5	60 (50.0)	44 (23.1)	104(33.5)
5–9	31 (25.8)	112 (58.9)	143(46.0)
10–14	17 (14.2)	31 (16.3)	48(15.5)
≥ 15	12 (10.0)	3 (1.5)	15(5.0)
Mean ± SD	6.8 ± 5.8	6.8 ± 3	6.82 ± 4.3
**Working hours per week**			
≤ 48 h	68 (56.7)	163 (85.8)	231(74.5)
> 48 h	52 (43.3)	27 (14.2)	79(25.4)
Mean ± SD	53.7 ± 16.8	44.0 ± 8.5	47.5 ± 13.7
**Physical Activity**			
High	23 (19.2)	63 (33.2)	86 (27.7)
Moderate	87 (72.5)	105 (55.3)	192 (61.9)
Low	10 (8.3)	22 (11.6)	32 (10.3)

A total of 73.2% (95%CI: 67.9–78.1) participants had MSD in the last 12 months, with approximately 41.6% (95%CI: 36.1–47.3) suffering from MSDs in the previous seven days of the survey. Almost one-fourth (28.1%, 95%CI: 23.1–33.4) of the participants accepted that musculoskeletal pain hampers normal activities. Only 15.8% (95%CI: 9.8–23.6) of doctors and 24.7% (95%CI: 18.8–31.5) of NOs took medical consultations for their musculoskeletal symptoms. Nearly half (47.1%, 95%CI: 41.4–52.8) of the participants suffered from MMS in the past 12 months. The highest number of WMS was associated with the axial region (54.2%), followed by the upper limb region (45.5%) and lower limb region (36.8%). Involvement of the upper limb region was significantly more among doctors (56.7%) than NOs (38.4%). During the past 12 months, 20.8% (95%CI: 13.9–29.2) of doctors and 18.4% (95%CI: 13.2–24.6) of NOs had WMS (MS in all three regions) (Table 2).

**Table 2:** Evaluation of MSDs among Health Care Professionals (HCPs) and their health-seeking behavior

Variables	Doctor (n = 120)	N.O.s (n = 190)	Total (n = 310)	P value
	No. (%)	No. (%)	No. (%)	
**Musculoskeletal symptoms (MS.)**				
Pain in the last 12 months	89 (74.2)	138 (72.6)	227 (73.2)	0.766
Pain in last 7 days	54 (45.0)	75 (39.5)	139 (41.6)	0.336
The problem in carrying out normal activities	31 (25.8)	56 (29.5)	87 (28.1)	0.487
Consulted physicians/taken any treatment	19 (15.8)	47 (24.7)	66 (21.3)	0.062
**Multisite musculoskeletal symptoms (MMS)**				
No MSD	36 (30.0)	53 (27.9)	89 (28.7)	0.254
One site	23 (19.2)	52 (27.4)	75 (24.4)	
Two or more sites	61 (50.8)	85 (44.7)	146 (47.1)	
**Widespread Musculoskeletal symptoms (WMS)**				
One region				
Axial	61 (50.8)	107 (56.3)	168 (54.2)	0.345
UL	68 (56.7)	73 (38.4)	141 (45.5)	**0.002**
LL	39 (32.5)	75 (39.5)	114 (36.8)	0.215
Two regions				
Axial and UL.	48 (40.0)	56 (29.5)	104 (33.5)	0.056
Axial and LL.	29 (24.2)	56 (29.5)	85 (27.4)	0.308
UL and LL.	31 (25.8)	40 (21.1)	71 (22.9)	0.329
Three regions	25 (20.8)	35 (18.4)	60 (19.4)	0.601

The lower back (49.7%) was the most affected site of all nine anatomical sites among healthcare professionals for MSD, whereas the elbow (6.8%) was the least affected site (see Supplementary file 1). Among doctors, neck pain (47.5%) was the most commonly affected site, followed by lower back (45.8%) and shoulder (30.8%) pain within the past 12 months. Among NOs, lower back pain (52.1%) was the most common site for MSDs, followed by neck (29.4%), ankle (24.7%), and knee (24.2%) pain (see Supplementary file 2).

**Fig. 2:**
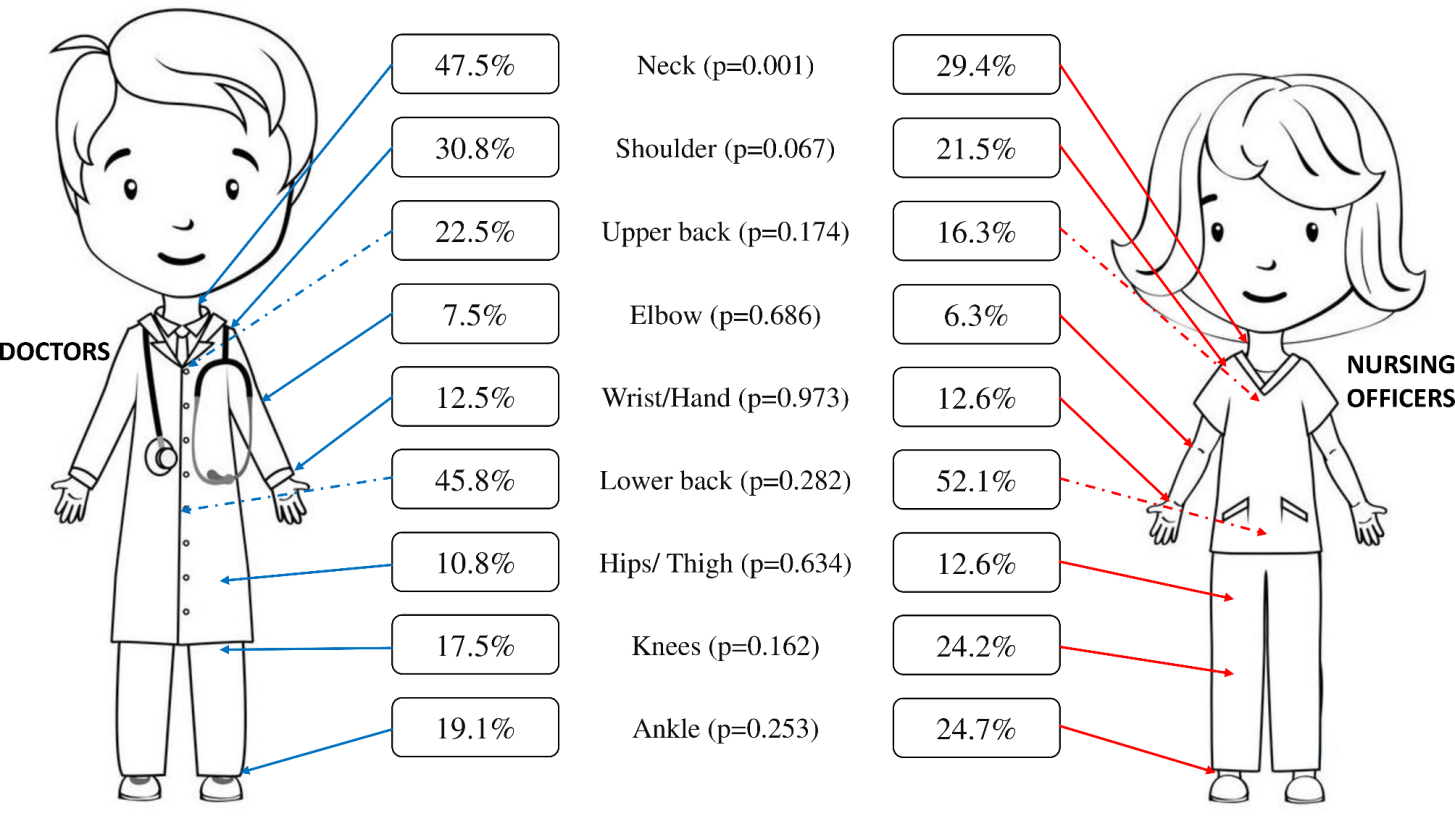
Distribution of MSD among doctors and nursing officers

A pictorial representation of the distribution of MSDs among healthcare professionals is illustrated in (Fig. 2).

**Fig. 3:**
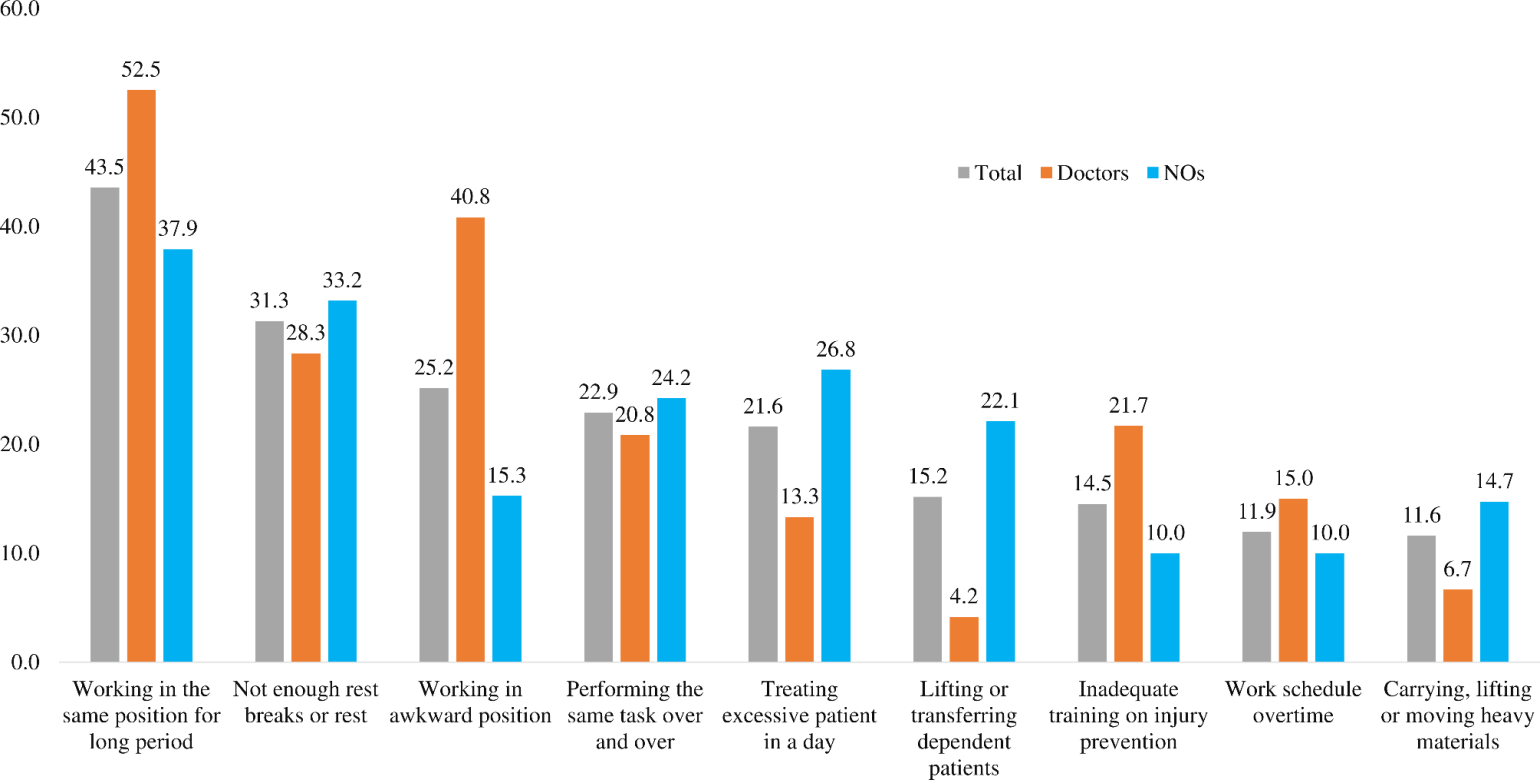
Risk factors of MSDs among doctors and nursing officers

Working in the same position for a long time (43.5%), not taking adequate breaks or rest (31.3%), working in an uncomfortable/awkward position (25.2%), performing the same task repeatedly (22.9%), and treating/handling more number of patients (21.6%) were the highest self-reported risk factors for MSDs among HCPs. (Fig. 3).

There was no statistically significant association of MSDs with age, marital status, work experience, daily sitting hours, and physical activity. Females had significantly higher odds of having pain in the upper back [aOR:2.49 (95%CI: 1.27–4.85)], neck [aOR:2.15 (95%CI: 1.22–3.77)], shoulder [aOR:2.8 (95%CI: 1.54–5.11)], hips [aOR:9.46 (95%CI: 3.95–22.68)] and knee [aOR:3.8 (95%CI: 1.99–7.26)]. Compared to doctors, the odds of developing neck pain were significantly [aOR:2.29 (95%CI: 1.2–4.36)] higher among NOs. Long duration of working hours (> 48 h per week) was a significant predictor of pain or discomfort in the upper back [aOR:2.24 (95%CI: 1.09–4.58)] and lower back [aOR:2.19 (95%CI: 1.23–3.99)]. Obese individuals (BMI > 25) were at a significantly higher risk of having pain in the hips region [aOR:2.83 (95%CI: 1.05–7.59)] (Table 3).

**Table 3:** Socio-demographic predictors of MSDs at different sites among healthcare professionals

Variables	Adjusted Odds Ratio with 95% CI								
	Upper back	Lower Back	Neck	Shoulder	Elbow	Wrist	Hips	Knee	Ankle
Age 30–39 yrs	1.08(0.52–2.34)	1.51(0.84–2.7)	1.55(0.83–2.93)	0.72(0.36–1.42)	1(0.29–3.38)	0.64(0.27–1.52)	2.17(0.78-6.0)	1.52(0.73–3.17)	0.84(0.43–1.65)
Age ≥ 40 yrs	1.05(0.23–4.83)	0.67(0.19–2.39)	0.78(0.22–2.73)	0.56(0.14–2.22)	1.97(0.27–14.52)	0.64(0.14–4.09)	4.7(0.66–33.41)	2.05(0.49–8.6)	0.31(0.05–1.73)
Female sex	**2.49(1.27–4.85)**	1.67(0.96–2.91)	**2.15(1.22–3.77)**	**2.8(1.54–5.11)**	2.1(0.76–5.81)	1.57(0.72–3.41)	**9.46(3.95–22.68)**	**3.8(1.99–7.26)**	1.83(0.98–3.44)
Married	0.78(0.37–1.66)	0.88(0.48–1.63)	0.61(0.32–1.15)	0.81(0.41–1.59)	0.74(0.22–2.49)	0.75(0.33–1.74)	0.96(0.36–2.59)	2.03(0.94-.62)	0.91(0.45–1.81)
Nursing officer	0.87(0.41–0.9)	0.56(0.31–1.05)	**2.29(1.2–4.36)**	1.47(0.73–2.97)	0.81(0.24–2.69)	1.01(0.42–2.55)	0.29(0.10–0.88)	0.43(0.20–0.94)	0.72(0.34–1.49)
> 48 working hrs per week	**2.24(1.09–4.58)**	**2.19(1.23–3.99)**	0.64(0.34–1.19)	1.63(0.85–3.12)	1.5(0.48–4.64)	1.4(0.59–3.34)	2.05(0.82–5.12)	0.85(0.41-.82)	1.75(0.89–3.44)
≥ 4 sitting hours per day	2.17(0.84–5.62)	0.49(0.26–0.92)	1(0.51–1.95)	0.98(0.47–2.03)	2.18(0.46–10.3)	0.52(0.23–1.2)	1.38(0.53-.83)	1(0.47–2.12)	0.83(0.42–1.66)
≥ 5 yrs work experience	1.14(0.53–2.45)	0.87(0.48–1.58)	1.46(0.76–2.8)	1.15(0.58–2.29)	2.78(0.73–10.66)	0.78(0.33–1.85)	0.68(0.25–1.88)	0.88(0.42–1.87)	1.4(0.69–2.84)
Moderate PA	0.52(0.18–1.55)	0.5(0.21–1.21)	0.86(0.34–2.18)	1.37(0.46–4.04)	0.67(0.14–0.5)	0.8(0.19–3.39)	3.51(0.81–5.38)	1.47(0.45–4.84)	1.73(0.57–5.31)
Low PA	0.83(0.33–2.11)	0.97(0.43–2.16)	1.15(0.5–2.62)	1.81(0.67–4.88)	1.52(0.31–7.49)	1.62(0.44–5.96)	1.53(0.38–6.22)	2.39(0.81–7.03)	2.04(0.72–5.79)
Overweight	0.88(0.37–2.11)	0.99(0.51–1.93)	1.14(0.56–2.32)	1.53(0.73-.33)	0.71(0.18–2.74)	1.77(0.66–4.74)	1.88(0.65-.91)	1.04(0.47–2.3)	1.17(0.53–2.55)
Obese	1.08(0.52–2.25)	1(0.56–1.8)	1.61(0.87-3.00)	1.45(0.73–2.89)	0.87(0.29–2.59)	1.43(0.58–3.55)	**2.83(1.05–7.59)**	0.9(0.44–1.83)	1.15(0.57–2.3)

Working in the same position for an extended period was the most common significant risk factor with Widespread Musculoskeletal symptoms (WMS), including axial [aOR:3.82 (2.15–6.80)], upper limb [aOR:3.12 (95%CI: 1.81–5.36)] and lower limb [aOR:3.78 (95%CI: 2.13–6.71)] regions. Risk factors like repeating the same task again and again [aOR:4.16 (95%CI: 1.63–10.60)] and lifting or transferring dependent patients [aOR:3.53 (95%CI: 1.19–10.47)] were also significantly associated with MSD involvement in the axial region. Working in an awkward position [2.68 (95%CI: 1.41–5.09)] and without having enough rest breaks [aOR:2.77 (95%CI: 1.41–5.43)] were found as significant risk factors for MSD involving the upper limb region. Exertion of treating more patients in a day was also a significant risk factor [aOR:3.44 (95%CI: 1.63–7.26)] for MSD in the lower limb region (Table 4). An association of risk factors (Unadjusted Odds Ratio) with different sites has been provided in Supplementary File 3.

**Table 4:** Associated risk factors for Widespread Musculoskeletal symptoms (WMS) among HCPs

Risk Factors	AOR (95%CI)		
	Axial	Upper limb	Lower limb
Working in an awkward position	1.71 (0.84–3.51)	**2.68 (1.41–5.09)**	0.51 (0.25–1.02)
Treating an excessive number of patients in a day	1.72 (0.68–4.38)	0.99 (0.45–2.15)	**3.44 (1.63–7.26)**
Working in the same position for a long period	**3.82 (2.15–6.80)**	**3.12 (1.81–5.36)**	**3.78 (2.13–6.71)**
Inadequate training in injury prevention	1.03 (0.38–2.78)	0.72 (0.29–1.74)	0.93 (0.39–2.24)
Performing the same task over and over	**4.16 (1.63–10.60)**	1.39 (0.66–2.92)	0.92 (0.44–1.92)
Not enough rest breaks or rest	2.02 (0.96–4.27)	**2.77 (1.41–5.43)**	1.51 (0.78–2.94)
Lifting or transferring dependent patients	**3.53 (1.19–10.47)**	1.10 (0.46–2.64)	2.07 (0.86–4.98)
Carrying, lifting, or moving heavy materials	0.65 (0.19–2.15)	1.73 (0.65–4.63)	1.01 (0.38–2.71)
Work schedule overtime	1.97 (0.61–6.29)	0.94 (0.36–2.45)	1.96 (0.78–4.92)

## Discussion

MSDs are one of the most important occupational health issues among healthcare professionals, mostly neglected. Published evidence on the burden of MSDs among HCPs and their risk factors and predictors is very limited from the Western part of India. This study aims to assess the prevalence, risk factors, and predictors of MSDs among healthcare professionals in a public health apex institution in the Western part of India. An assessment of the associated ergonomic and biomechanical risk factors and evaluation of sites involved in MSDs through this study can fill the gap in baseline data for developing intervention strategies for HCPs in the future. The socio-demographic and work-related attributes of doctors and NOs in the present study were more or less similar to the other published studies conducted on healthcare workers in several healthcare facilities for the assessment of MSDs in India [6, 11, 12, 19].

In our study, about three fourth of the HCPs reported having musculoskeletal pain at different sites in the last 12 months, and nearly 40% stated having the same in the last week (point prevalence). This finding is within range of the prevalence reported by the published scientific studies on healthcare workers from different parts of the country [6, 10–14]. A systematic review reflected an alarming state where the prevalence of MSD among handicraft workers was nearly 38.5-100% [20]. This prevalence is also comparable to the prevalence of MSDs reported among manual harvesting farmers of Rajasthan (77.9%) [21] and construction workers (77%) in Andhra Pradesh [22]. Surprisingly, this burden is higher than the prevalence of MSDs in railway sahayaks/coolies (65%) [23] and industrial workers (59.4%) [24], and the general population (25.9%) in India [25]. This high burden of MSDs among HCPs compared to heavy workers and general people raises concerns regarding the ergonomics of the work environments in Indian healthcare facilities. A study in Saudi Arabia reflected that around 92% of respondents have developed musculoskeletal pain after joining the physiotherapy profession [26]. Further, the prevalence of work-related lower back pain among physical therapists in Riyadh was high, affecting patient care and daily activities of the therapists [27]. Further, a study conducted in Uganda reflect that there were significant differences reported in MSD among nursing staff across different hospital settings which were worse in the public hospitals as compared to the private and private not for profit hospitals (p < 0.001) [28].

Unlike the study by Yasobant S et al. [6], the differences for the 12 months and the point prevalence of MSDs among doctors and nurses, were insignificant in the present study. Almost half of the HCPs reported multisite musculoskeletal pain/symptoms. This is relatively lower than the multisite involvement findings reported by Chuan Lin S et. at. [29] (86.2%) and Nguyen T.H. et al. [30](90%), but higher than the findings reported by Kumar M. et al. [31]. (33.1%) and Rahman M et al. (42.2%) [32]. During the past 12 months, almost 20% of HCPs had WMS (MS in all three regions) in our study. This is almost similar (17.1%) to the WMS reported during the past 12 months among district hospital nurses in Haiphong, Vietnam [30].

In our study, the highest prevalence of MSDs was in the lower back (49.7%) and neck (36.5%) in the last 12 months, and the odds of developing neck pain were significantly higher among NOs. This finding aligns with other studies done in India [13, 14, 33]. Specialists in orthopedics complained of pain only in the lower back region, but specialists in neurology also reported pain in the buttocks, thighs, and legs, as well as the lower back region [27]. Similar to the present study, many other studies have reported variations in the most common site of MSDs among doctors and nurses. These differences in the sites may be attributed to the differences in the study sites and sampling methods, which may lead to variations in the socio-demographic and ergonomic risk factors of MSDs [3, 6, 12, 14, 32, 34, 35].

Similar to other published studies, females had higher odds of developing MSDs in the present study. In line with this finding, the prevalence of WRMDs after joining the dental profession was high in female professionals compared with their male counterparts in a study [12, 32]. This preponderance of MSDs among women may be because most of them in India are overworked due to additional home obligations that prevent them from getting enough rest breaks. Biological variables include susceptibility to obesity, age-related bone changes after menopause, physical changes following birth, and differences in natural build-up increase their risk of acquiring MSDs [30, 36, 37].

In our study, long working hours (> 48 h per week) were found as a significant predictor of musculoskeletal pain in the upper and lower back. In the dental profession, work-related disorders have a major effect on their daily activities other than work, especially in those with patient contact of more than 30 h a week [38]. Pain at other sites was not significantly associated with working hours. Many authors also found no significant association between working hours and a 12-month prevalence of WRMSDs [39]. Obese individuals (BMI > 25) were found at a significantly higher risk of having pain in the hips region. The Association between obesity and pain in the hip region is supported by scientific evidence [40, 41]. Working in the same position for a long time, not having enough rest breaks, working in an awkward position, performing the same tasks repeatedly, and having a higher patient load were the most common risk factors reported by HCPs in the present study. Many studies have reported similar occupational risk factors for MSDs among healthcare workers in and outside India [28, 42].

Our study brings out a few implications in the prevention of MSD in health sector. As epidemiological data has demonstrated that occupational risk factors such as awkward postures, highly repetitive activities or handling heavy loads are among the risk factors that studies have shown to damage the bones, joints, muscles, tendons, ligaments, nerves and blood vessels, leading to fatigue, pain and WMSDs. The Karsh model (2006) provides a framework to assess the factors relating to the workplace that determine exposure to WMSD risk factors i.e., the work organization, the socio-cultural context, and the environment surrounding the workplace [43]. Thus, health facility designing must be done according to ergonomic requirements. Ideally, adaptations are made to the furniture, equipment, and tools used by the participants and the work environment to enable them to perform adequately without risk to himself/herself, co-workers, and the public. It is also necessary to improve the worker’s adaptation to the job through, for example, special training and the use of personal protective equipment. Furthermore, there has been increasing recognition of “Psychosocial factors” after the COVID-19 pandemic. The possible mechanism of these psychosocial factors and WMSD has been elaborated well by Sauter and Swanson (1995) [44].

Rest breaks can be vital in reducing MSDs from fatigue and long working schedule. This could be a tea or lunch break. Further, small time day/night shifts with rotation could be a step in this direction. A study conducted on university student during homestay during the COVID-19 period in Rajasthan found that physical activity intervention (PAI) on computer users reduces the risk of MSDs in the long term for different body regions [45]. Thus, various activities like yoga, stretching exercises, meditation, sports, physiotherapies, and music can help in breaking the vicious cycle of long, extended working hours Along with this, a model-based health education intervention has promising results in improving ergonomic posture in computer workers [46]. This may be incorporated as part of the training process during the recruitment of health workers.

One limitation of our study was that the data was collected from only one apex institution, which may limit the generalizability of the findings. But, at the same time, this evidence may support filling up the gap about the prevalence of MSDs among Doctors and Nursing Officers in Western Rajasthan.

## Conclusions

Our study findings revealed that three-fourths of doctors, and nursing officers had MSDs. In a nutshell, females were predominantly at greater risk of MSDs for developing MSD, and this was multiplied in women who are NOs, work for > 48 hours per week, and fall in the obese category. Working in an awkward position, treating an excessive number of patients in a day, working in the same position for a long period, performing the same task over and over, not having enough rest breaks or rest, and lifting or transferring dependent patients were identified as significant risk factors for WRMSDs among HCPs. Workplace ergonomics and environment have immense potential to reduce the prevalence of MSDs, especially in overstrained doctors and nursing officers in Indian healthcare settings.

## Electronic supplementary material

Below is the link to the electronic supplementary material.

Supplementary Material 1

Supplementary Material 2

Supplementary Material 3

## Data Availability

All data generated or analysed during this study are included in this published article [and its supplementary information files].

## Abbreviations

aOR: Adjusted Odds Ratio
ARC: American College of Rheumatology
CI: Confidence Interval
DALY: Disability-Adjusted Life Years
GoI: Government of India
HCDS: Health Care Delivery System
IPHQ: International Physical Activity Questionnaires
MMS: Multisite Musculoskeletal Symptoms
MS: Musculoskeletal Symptoms
MSDs: Musculoskeletal disorders
NMQ: Nordic Musculoskeletal Questionnaire
NOs: Nursing Officers
WHO: World Health Organization
WMS: Widespread Musculoskeletal Symptoms
WRMSDs: Work Related Musculoskeletal disorders
