# Supplementary material for: Musculoskeletal disorders among doctors and nursing officers : an occupational hazard of overstrained healthcare delivery system in western Rajasthan, India
Source: BMC Musculoskelet Disord. 2023 May 4;24:349. doi: 10.1186/s12891-023-06457-z (PMC10157123; doi:10.1186/s12891-023-06457-z)
Supplement: Supplementary file 1 — Supplementary Material 1 [file 12891_2023_6457_MOESM1_ESM.docx]

**Supplementary Table 2. Status of MSDs among Nursing Officers and their health-seeking behavior (n=190)**

|  | **Pain in the last 12 months**  **No. (%)** | **Pain in the last seven days**  **No. (%)** | **The problem in carrying out normal activities**  **No. (%)** | **Consulted physician**  **No. (%)** |
| --- | --- | --- | --- | --- |
| Neck | 56 (29.4) | 21 (11.0) | 16 (8.4) | 8 (4.2) |
| Shoulder | 41 (21.5) | 19 (10.0) | 14 (7.3) | 9 (4.7) |
| Upper back | 31 (16.3) | 14 (7.3) | 7 (3.6) | 9 (4.7) |
| Elbow | 12 (6.3) | 3 (1.5) | 6 (3.1) | 4 (2.1) |
| Wrists/ hands | 24 (12.6) | 4 (2.1) | 8 (4.2) | 5 (2.6) |
| Lower back | 99 (52.1) | 42 (22.1) | 36 (18.9) | 25 (13.1) |
| Hips/thighs | 24 (12.6) | 12 (6.3) | 9 (4.7) | 6 (3.1) |
| Knees | 46 (24.2) | 27 (14.2) | 19 (10.0) | 11 (5.7) |
| Ankles/feet | 47 (24.7) | 23 (12.1) | 17 (8.9) | 13 (6.8) |
