# Supplementary material for: Musculoskeletal disorders among doctors and nursing officers : an occupational hazard of overstrained healthcare delivery system in western Rajasthan, India
Source: BMC Musculoskelet Disord. 2023 May 4;24:349. doi: 10.1186/s12891-023-06457-z (PMC10157123; doi:10.1186/s12891-023-06457-z)
Supplement: Supplementary file 2 — Supplementary Material 2 [file 12891_2023_6457_MOESM2_ESM.docx]

**Supplementary table 1: Status of MSDs among doctors and their health-seeking behavior (n=120)**

|  | **Pain in the last 12 months**  **No. (%)** | **Pain in the last seven days**  **No. (%)** | **The problem in carrying out normal activities**  **No. (%)** | **Consulted physician**  **No. (%)** |
| --- | --- | --- | --- | --- |
| Neck | 57 (47.5) | 15 (12.5) | 15 (12.5) | 8 (6.6) |
| Shoulder | 37 (30.8) | 15 (12.5) | 12 (10.0) | 8 (6.6) |
| Upper back | 27 (22.5) | 7 (5.8) | 8 (6.6) | 4 (3.3) |
| Elbow | 9 (7.5) | 2 (1.6) | 2 (1.6) | 2 (1.6) |
| Wrists/ hands | 15 (12.5) | 6 (5.0) | 2 (1.6) | 4 (3.3) |
| Lower back | 55 (45.8) | 20 (16.6) | 14 (11.6) | 7 (5.8) |
| Hips/thighs | 13 (10.8) | 6 (5.0) | 6 (5.0) | 2 (1.6) |
| Knees | 21 (17.5) | 8 (6.6) | 4 (3.3) | 5 (4.1) |
| Ankles/feet | 23 (19.1) | 6 (5.0) | 6 (5.0) | 5 (4.1) |
