# Supplementary material for: Musculoskeletal disorders among doctors and nursing officers : an occupational hazard of overstrained healthcare delivery system in western Rajasthan, India
Source: BMC Musculoskelet Disord. 2023 May 4;24:349. doi: 10.1186/s12891-023-06457-z (PMC10157123; doi:10.1186/s12891-023-06457-z)
Supplement: Supplementary file 3 — Supplementary Material 3 [file 12891_2023_6457_MOESM3_ESM.docx]

**Supplementary Table 3: Association of risk factors with sites**

| **Risk Factors** | **Unadjusted Odds Ratio (95%CI)** | | | | | | | | |
| --- | --- | --- | --- | --- | --- | --- | --- | --- | --- |
|  | **Neck** | **Shoulder** | **Upper back** | **Elbow** | **Wrist** | **Lower back** | **Hips** | **Knee** | **Ankle** |
| Working in an awkward position | 4.11(2.40-7.04) | 2.71(1.56-4.71) | 3.74(2.30-6.08) | 1.65(0.87-3.11) | 1.86(1.09-3.19) | 3.07(1.87-5.06) | 2.78(1.48-5.23) | 2.43(1.20-4.92) | 1.57(0.78-3.13) |
| Treating an excessive patient in a day | 2.6(1.49-4.52) | 3.59(2.02-6.39) | 5.84(3.28-10.4) | 2.03(1.04-3.96) | 5.73(3.22-10.2) | 4.37(2.54-7.52) | 2.62(1.37-5.00) | 3.12(1.53-6.38) | 4.36(2.15-8.86) |
| Working in the same position for a long period | 2.14(1.16-3.93) | 4.18(2.26-7.74) | 3.67(1.99-6.77) | 2.58(1.28-5.2) | 4.54(2.46-8.35) | 4.25(2.34-7.71) | 2.72(1.37-5.40) | 1.81(0.82-4.00) | 4.81(2.33-9.95) |
| Inadequate training on injury prevention | 2.95(1.20-7.26) | 4.58(1.85-11.31) | 4.57(1.63-12.82) | 3.3(1.25-8.71) | 8.14(3.14-21.1) | 2.6(1.06-6.34) | 3.94(1.54-10.13) | 5.74(2.19-15.03) | 4.32(1.62-11.53) |
| Performing the same task over and over | 1.58(0.77-3.26) | 3.42(1.69-6.9) | 3.9(1.86-8.17) | 2.33(1.04-5.18) | 4.00(1.99-8.03) | 5.63(2.75-11.56) | 3.00(1.39-6.45) | 3.87(1.72-8.69) | 3.7(1.65-8.29) |
| Not enough rest breaks or rest | 2.97(1.72-5.13) | 5.94(3.08-11.46) | 5.63(3.43-9.22) | 3.73(1.82-7.69) | 9.58(4.67-19.62) | 6.26(3.59-10.94) | 5.35(2.49-11.51) | 2.22(1.07-4.61) | 3.63(1.65-7.98) |
| Lifting or transferring dependent patients | 1.51(0.72-3.16) | 4.33(2.12-8.85) | 3.56(1.69-7.49) | 2.12(0.92-4.85) | 4.49(2.20-9.14) | 3.4(1.69-6.87) | 3.81(1.77-8.18) | 3.52(1.53-8.07) | 3.37(1.48-7.72) |
| Carrying, lifting or moving heavy materials | 1.12(0.61-2.07) | 3.43(1.89-6.21) | 2.94(1.68-5.16) | 1.81(0.90-3.63) | 2.79(1.55-5.03) | 2.71(1.55-4.74) | 2.41(1.24-4.72) | 1.99(0.94-4.23) | 1.9(0.90-4.02) |
| Work schedule overtime | 1.26(0.69-2.28) | 5.4(2.99-9.78) | 4.98(2.76-8.99) | 1.91(0.96-3.80) | 2.56(1.43-4.59) | 3.36(1.94-5.85) | 3.53(1.84-6.78) | 2.48(1.19-5.15) | 4.59(2.25-9.37) |
